# A Prognostic Analysis of the Outcomes in Patients With Anti-γ-Aminobutyric Acid B Receptor Encephalitis

**DOI:** 10.3389/fimmu.2022.847494

**Published:** 2022-04-19

**Authors:** Weibi Chen, Yunyun Wang, Xiaoyuan Guo, Lehong Gao, Zhaoyang Huang, Yicong Lin, Qin Xue, Gang Liu, Yan Zhang, Yingying Su

**Affiliations:** ^1^Xuanwu Hospital, Capital Medical University, Beijing, China; ^2^Department of Neurology, China-Japan Friendship Hospital, Beijing, China; ^3^Dongzhimen Hospital, Beijing University of Chinese Medicine, Beijing, China

**Keywords:** autoimmune encephalitis (AE), GABA_B_R, Encephalitis (MeSH), prognosis, tumor

## Abstract

**Objective:**

To evaluate neurological function and its influencing factors in patients with anti-γ -aminobutyric acid B receptor (GABA_B_R) encephalitis.

**Methods:**

This was a clinical cohort study of patients diagnosed with anti-GABA_B_R encephalitis; long-term follow-up was performed by telephone. Clinical factors associated with prognosis were analyzed, including clinical manifestations, laboratory examinations, imaging features, tumor comorbidities and therapeutic responses.

**Results:**

Twenty-two patients with anti-GABA_B_R encephalitis were evaluated (median age: 55 years). Lung cancer was detected in eight patients. All were with serum tumor markers (mainly NSE), and three of them had additional onconeuronal antibodies. The patients with tumors were older than the patients without tumors and more likely to develop status epilepticus (62.5% vs. 14.3%; p = 0.052), central hypoventilation (50% vs. 7.1%; p = 0.039), and hyponatremia (87.5% vs. 14.3%; p = 0.001). The patients with tumors had higher mortality (87.5% vs. 0%; p < 0.05). Although 92.9% of the patients without tumors became functionally independent (mRS ≤2), sequelae of symptomatic seizures, neuropsychiatric symptoms, and cognitive impairment were still observed in 14.3%, 21.4%, and 21.4% of patients, respectively.

**Conclusions:**

(1) Elderly patients with anti-GABA_B_R antibodies, especially those with severe symptoms, serum tumor markers, and additional onconeuronal antibodies, should be screened for lung cancer. (2) Anti-GABA_B_R encephalitis with tumors has a poor prognosis. (3) Most patients without tumors achieve self-care, but some still experience remaining neurological deficits.

## Introduction

With the finding of autoantibodies, cases of encephalitis that were previously presumed to be viral or idiopathic in etiology have been determined to be caused by autoimmune mechanisms. Moreover, these autoantibodies can also be used to distinguish autoimmune encephalitis (AE) in various subgroups with distinct clinical phenotypes and different prognoses (1, 2). Anti-γ-aminobutyric acid B receptor (GABA_B_R) encephalitis is one form of AE that is caused by autoantibodies targeting GABA_B_Rs and was first reported in 2010 by Lancaster et al. (3). It is a relatively rare disease, accounting for approximately 5% of all cases of autoimmune synaptic encephalitis (4). Most of the previously described patients had typical features of limbic encephalitis (LE) such as seizures, memory deficits or behavioral problems, which were mainly related to the inhibition of the high-density expression of GABA_B_R in the hippocampus (5). A subset of patients also present with cerebellar ataxia (6) and opsoclonus-myoclonus (7). Additionally, GABA_B_R autoimmunity likely progresses to severe symptoms of status epilepticus and consciousness disturbance, and severe complications such as respiratory failure; therefore, these patients require extensive critical care management (3). Previous studies showed that the fatality rates of this disorder range between 22% and 45% (3, 8–12).

It has been reported that lung cancer or tumors of neuroendocrine origin may be observed in patients with anti-GABA_B_R encephalitis. However, there are limited data about the difference in functional outcomes between patients with paraneoplastic and idiopathic anti-GABA_B_R encephalitis or the correlation between the clinical manifestations and neurological outcomes. To further identify the potential factors that affect these outcomes, we analyzed clinical features, laboratory evaluation results, imaging features, electroencephalogram (EEG) features, and treatment responses. These data may help promote recognition of the disorder, which is crucial for improving oncologic and neurologic outcomes.

## Methods

### Study Participants and Setting

Anti-GABA_B_R encephalitis patients who were consecutively admitted to the Department of Neurology at Xuanwu Hospital Capital Medical University between December 2011 and January 2020 were enrolled in this cohort study. The inclusion criteria were as follows: (1) anti-GABA_B_R encephalitis was diagnosed based on the following criteria (13): 1) positive results of GABA_B_R antibodies in serum or cerebral spinal fluid (CSF); the presence of anti-GABA_B_R antibodies was measured using fixed cell-based indirect immunofluorescence test (IIFT) kits (Euroimmun AG, Lübeck, Germany). Other antibodies were also examined by IIFT, including antibodies against neuronal cell surface antigens N-methyl-D-aspartate (NMDA) receptor, a-amino-3-hydroxy-5-methyl-4-isoxazol-propionic acid (AMPA) receptors 1 and 2, contactin associated protein 2 (CASPR2), leucine-rich gliomainactivated protein 1 (LGI1), as well as antibodies against intracellular neuronal antigens Hu, Ri, Yo, glutamic acid decarboxylase 65 (GAD65), amphiphysin, collapsin response mediator protein (CV2), sry-related box genes (SOX1) and paraneoplastic antigen Ma2 (PNMA2). 2) clinical data indicative of LE, which was defined as encephalitis with predominant clinical involvement of the limbic system (subacute onset of working memory deficits, seizures, or psychiatric symptoms) or magnetic resonance imaging (MRI) fluid-attenuated inversion recovery (FLAIR)/T2 abnormalities in the medial temporal lobes. (2) Other disorders were reasonably excluded. (3) Informed consent was obtained from the patient’s family. The exclusion criteria were as follows: (1) the patient was lost to follow-up; and (2) the patient had cognitive deficits or epilepsy prior to the onset of anti-GABA_B_R encephalitis. This study was approved by the Ethics Committee of Xuanwu Hospital, Capital Medical University (2020-104).

### Data Collection

A uniformly designed form was used to record patient demographics, clinical characteristics, laboratory results, imaging findings, EEG data, tumor association, immunotherapy selection and therapeutic responses. Tumor screenings, such as CT thorax/abdomen, were conducted for all patients with anti-GABABR encephalitis. In 22 patients enrolled in this study, positron emission tomography/computed tomography (PET/CT) was performed in 17 (77%), and of 16 patients who were tumor negative on CT thorax/abdomen, 11 (69%) underwent PET/CT. Eight serum tumor markers, including alpha-fetoprotein (AFP), neuron specific enolase (NSE), cytokerantin-19-fragment (Cyfra21-1), carbohydrate antigen (CA)125, CA199, CA153, CA72-4, and carcinoembryonic antigen (CEA), were also tested in all. Based on tumor comorbidity, the patients were divided into two groups: a group with tumors and a group without tumors. Additionally, based on the severity of clinical symptoms [with coma (Glasgow Coma Scale ≤ 8), status epilepticus, or mechanical ventilation] or admission to the intensive care unit (ICU), the patients were divided into two groups: a nonsevere group and a severe group. Moreover, based on the antibody titers in the CSF, the patients were divided into two groups: the low-titer group and the high-titer group. The low-titer group included patients who were with titer of GABA_B_R antibodies equal to or less than 1:32 in the CSF. The high-titer group included patients who were strongly positive [titer of 1:100 and above] for GABA_B_R antibodies in the CSF. Immunotherapeutic methods and therapeutic responses were also recorded.

### Treatment and Prognosis Evaluation

All patients received tumor screening, symptomatic treatment, and immunotherapy. Immunotherapy included first-line therapy [glucocorticoid therapy (5 d, IV methylprednisolone, followed by oral prednisolone), IV immunoglobulin (5 d, 0.4 g/kg), or plasma exchange], and second-line immunosuppressants (cyclophosphamide, rituximab, or mycophenolate mofetil) when necessary.

Since it was not easy to assess residual cognitive function in a large geographically dispersed population or in patients whose functional status prevented clinical visits, telephone interviews were conducted. For the enrolled patients, neurological outcomes, including motor disability (modified Rankin Scale, mRS), quality of daily life (activities of daily living, ADL), remote symptomatic epilepsy, neuropsychiatric symptoms (neuropsychiatric inventory, NPI), and cognitive ability (modified telephone interview for cognitive status, TICS-M), were evaluated. For the patients whose condition could not be clearly investigated by telephone follow-up, a clinical interview was also added. mRS scores included a motor disability scale ranging from 0 for no symptoms to 6 for death. The scale was divided into good (mRS ≤2) and poor (mRS ≥3) outcomes. The ADL instrument is a 14-item (4 points for each item) scale used to evaluate the activities of daily life that included six items for basic physical self-maintenance (PSM) and eight items for instrumental ADL (IADL). The Neuropsychiatric Inventory (NPI) describes 12 behavioral symptoms, including delusions, hallucinations, agitation, depression, anxiety, euphoria, apathy, disinhibition, irritability, aberrant motor behavior, sleep behavior and appetite changes, and can be used to assess neuropsychiatric symptoms (NPSes). NPSes were considered to exist if the NPI scores were ≥ 1. The TICS-M (50 points) is a 13-item cognitive screening instrument. It includes items sensitive to amnestic mild cognitive impairment (delayed recall), which has been reported as a sensitive and specific indicator for dementia. Cognitive impairment was defined as a TICS-M score lower than the cutoff value of 34 for mild cognitive impairment.

### Statistical Analysis

Statistical analyses were performed with the statistical software SPSS 22.0 (IBM Corporation, Armonk, NY, USA). Patient characteristics were summarized by expressing categorical variables as counts (proportions) and continuous variables as medians (interquartile ranges, IQRs). Differences among groups were studied using Fisher’s exact tests and Mann–Whitney U tests with a significance level of 0.05.

## Results

### Clinical Features and Ancillary Examination Investigation

With the exception of four patients who were lost to follow-up and excluded from this study, thirteen males and nine females, with an average age of 55 years, were evaluated. A summary of the clinical information of the 22 patients with anti-GABA_B_R encephalitis is presented in **Table 1**. In this study, the most characteristic presentation was seizures, which occurred at diagnosis in 100% of patients and presented as the initial symptom in 81.8% of patients. Other limbic symptoms characterized by memory deficits and psychiatric disorders were also common. Moreover, in 68.2% of the 22 patients, the clinical diagnosis of LE was confirmed by MRI findings of increased unilateral (5 patients) or bilateral (10 patients) FLAIR/T2 signal abnormalities in the medial temporal lobes. EEG data were available for 21 patients; the findings were unremarkable in 5 patients and showed generalized or focal slowing in the remaining 16 patients. Among these 16 patients, 56.3% (9/16) had foci of epileptic activity. All patients in this study underwent lumbar puncture. GABA_B_R antibodies were found in 100% of CSF samples and in 86.4% of serum samples.

**Table 1:** Clinical features of 22 patients with anti- GABA_B_R encephalitis and comparison between the group with tumors and without tumors.

Variable	Total	Tumor comorbidity		p Value
	(N =22)	Yes (N = 8)	No (N = 14)	
Male, n (%)	13 (59.1%)	5 (62.5%)	8 (57.1%)	1.000
Age at onset, y, x ± s	55.3 ± 10.2	60.3 ± 4.3	52.2 ± 1.6	0.037
Days between onset and immunotherapy, d, median (IQR)	24.5 (20.0-51.0)	22.5 (18.5-28.3)	45 (22.3-90)	0.04
Symptoms at diagnosis, n (%)				
Status epilepticus	7 (31.8%)	5 (62.5%)	2 (14.3%)	0.052
Memory deficit	21 (95.5%)	8 (100%)	13 (92.9%)	1.000
Psychiatric disorders	16 (72.7%)	7 (87.5%)	9 (64.3%)	0.351
Disturbance of consciousness	8 (36.4%)	4 (50%)	4 (28.6%)	0.384
Central hypoventilation	5 (22.7%)	4 (50%)	1 (7.1%)	0.039
Hyponatremia, n (%)	9 (45%)	7 (87.5%)	2 (14.3%)	0.001
CSF findings, n (%)				
Cell count>5cells/ul	8 (36.4%)	4 (50%)	4 (28.6%)	0.386
Protein>0.45g/l	7 (31.8%)	2 (25%)	5 (35.7%)	1.000
GABA_B_R antibody positive				0.662
High-level	9 (40.9%)	4 (50%)	5 (35.7%)	
Low-level	13 (59.1%)	4 (50%)	9 (64.3)	
OB positive, n (%)	10/19 (52.6%)	4/7 (57.1%)	6/12 (50%)	1.000
Tumor markers	13 (59.1%)	8 (100%)	6 (42.9%)	0.018
Paraneoplastic antibody	3 (37.5%)	3 (37.5%)	0 (0%)	0.036
MRI, n (%)				1.000
Normal or unremarkable	7 (31.8%)	3 (37.5%)	4 (28.6%)	
Hyperintensity of the medial temporal lobes	15 (68.2%)	5 (67.5%)	10 (71.4%)	
Last mRS				0.000
1-2	13 (59.1%)	0 (0%)	13 (92.9%)	
>2	9 (40.9%)	8 (100%)	1 (7.1%)	
Death	7 (31.8%)	7 (87.5%)	0 (0%)	0.000

GABA, γ-aminobutyric acid; IQR, interquartile range; CSF, cerebrospinal fluid; OB, oligoclonal bands; MRI, magnetic resonance imaging; mRS, modified Rankin Scale.

### Treatment and Outcomes

**Figure 1:**
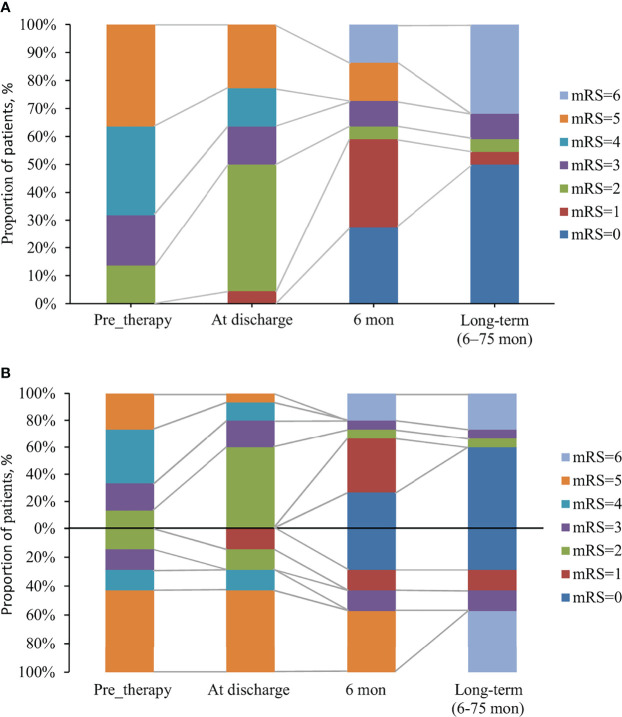
The changes of mRS over time in anti-GABA_B_R encephalitis patients with and without response to first-line immunotherapy. **(A)** The changes of mRS in 22 patients with anti-GABA_B_R encephalitis. **(B)** 22 patients were divided into two groups: a group with response to first-line immunotherapy (above the x-axis, n = 15) and a group with no response (below the x-axis, n = 7), based on the effects of first-line immunotherapy.

Overall, 31.8% (7/22) of patients exhibited resistance to the initial immunotherapy, including 50% (4/8) of patients with tumors and 21.4% (3/14) of patients without tumors (p =0.343). The median follow-up period with these 22 patients of anti-GABA_B_R encephalitis was 13 months (range: 6–75). The mRS scores ranged from 2-5 (median 4) during acute illness, and the changes of mRS over time in patients with and without response to first-line immunotherapy were shown in **Figure 1**. Seven patients with lung cancer died during follow-up, and 13 (59.1%) had favorable outcomes during the recovery phase. The functional outcomes of the 15 survivors with anti-GABA_B_R encephalitis are shown in **Table 2**. At the last follow up, the median ADL score of these patients was 14 points and the median TICS-M score was 35 points. The median follow-up period for them was 42 months (range: 8–75). Eleven patients had legacy symptoms, including symptomatic epilepsy (20%), neuropsychiatric symptoms (26.7%) and cognitive impairment (26.7%).

**Table 2:** Functional outcomes of living patients with anti-GABA_B_R encephalitis at the last follow-up.

Outcome Variable	Number (%) (N = 15)
Sequelaes, n (%)	
Symptomatic epilepsy	3 (20%)
NPSes	4 (26.7%)
Cognitive impairment	4 (26.7%)
ADL in all, median (IQR)	14 (14-16)
PSM	6 (6-6)
IADL	8 (8-10)
TICS-M in all (0-50), median (IQR)	35 (31-37)
TICS-M memory in all (0-20)	9 (7-11)
TICS-M instant memory (0-10)	5 (4-6)
TICS-M delayed recall (0-10)	4 (2-5)
TICS-M compute (0-5)	4 (2-5)

IQR, interquartile range; mRS, modified Rankin Scale; NPSes, Neuropsychiatric symptoms; ADL, activities of daily living; PSM, physical self-maintenance; IAD, instrumental ADL; TICS-M, modified telephone interview for cognitive status.

### Outcome Comparisons Between Groups With and Without Tumors

Overall, our study found that 36.4% (8/22) of patients had tumors, and all of these patients were found to have lung cancers. Among these eight patients, seven of them had small cell lung cancer (SCLC) and the other one with bone metastasis did not have pathologic confirmation. As shown in **Table 1**, the median age of the group with tumors was older than that of the group without tumors [60 (range: 55-69) vs. 52 (range: 33-77), P=0.037]. Individuals with neoplastic anti-GABA_B_R encephalitis were more likely to develop status epilepticus (p = 0.052), central hypoventilation (p = 0.039), and hyponatremia (p = 0.001). Moreover, significantly higher levels of serum tumor markers were found in this cohort (p = 0.018). Among them, NSE was the most common tumor markers, which was high in 50% (4/8) of the group with tumors and 14% (2/14) of the group without tumors, respectively. Three patients with SCLC had additional onconeuronal antibodies in serum: to Hu in two patients and to CV2 in one patient. Although patients harboring an underlying tumor had a shorter time to immunotherapy initiation, they had a tendency toward higher mortality (87.5% vs. 0%; p < 0.05). In eight patients with lung cancer, seven died within 1–12 months due to neoplastic complications, and the remaining patient survived but did not achieve self-care (mRS = 3), who had the sequelaes of symptomatic seizures, neuropsychiatric symptoms, cognitive impairment (TICS-M=13) and decreased quality of daily life (ADL=39). Inspiringly, none of the patients without tumors had died by the last follow-up, and 92.9% of these patients became functionally independent (mRS ≤ 2). However, sequelaes of symptomatic seizures, neuropsychiatric symptoms, and cognitive impairment persisted in 14.3%, 21.4%, and 21.4% of these patients, respectively.

### Comparison of Outcomes Between Groups With Severe and Nonsevere Symptoms

The incidence of severe symptom status epilepticus, coma and central hypoventilation was 31.8%, 31.8% and 22.7%, respectively. Eleven patients required intensive care, and the patients were then divided into a nonsevere group (11) and a severe group (11). The clinical characteristics and prognosis of anti-GABA_B_R encephalitis patients between the two groups are shown in **Table 3**. The number of CSF cells was significantly higher in the severe group than in the nonsevere group (p < 0.05). Although there was a tendency of longer hospital stays in the severe group (p = 0.051), no significant differences between the two groups were found in terms of demographics, main accessory examinations or functional outcomes.

**Table 3:** Comparison between anti-GABA_B_R encephalitis patients with two levels of severities of clinical symptoms, and with two titer levels of anti-GABA_B_R antibodies.

Variable	Clinical severity		p Value	Titer of antibody		p Value
	Non-severe group (N = 11)	Severe group (N = 11)		High-level (N =9)	Low-level (N =13)	
Male, n (%)	6 (54.5%)	7 (63.6%)	1.000	6 (66.7%)	5 (53.8%)	0.674
Age at onset, y, x ± s	53.4 ± 12.5	57.2 ± 7.4	0.395	60.2 ± 11.6	51.9 ± 7.9	0.057
Serum positive for GABA_B_R antibody, n (%)	11 (100%)	10 (90.9%)	1.000	9 (100%)	12 (92.3%)	1.000
CSF findings, n (%)						
Cell count>5cells/ul	1 (9.1%)	7(63.6%)	0.024	5 (55.6%)	3 (23.1%)	0.187
Protein>0.45g/l	2 (18.2%)	5 (45.5%)	0.361	5 (55.6%)	2 (15.4%)	0.074
OB positive^†^, n (%)	4 (40%)	6 (66.7%)	0.370	5 (62.5%)	5 (45.5%)	0.650
MRI, n (%)			1.000			1.000
Normal or unremarkable	3 (27.3%)	4 (36.4%)		3 (33.3%)	4 (30.8%)	
Hyperintensity of the medial temporal lobes	8 (72.3%)	7 (63.6%)		6 (66.7%)	9 (69.2%)	
Length of hospital stay, d, median (IQR)	13 (10-20)	17 (14-35)	0.051	17 (13-23)	14 (11.5-21.5)	0.663
Last mRS			1.000			0.384
1-2	7 (63.6%)	6 (54.5%)		4 (44.4%)	9 (69.2%)	
>2	4 (36.4%)	5 (45.5%)		5(55.6%)	4 (30.8%)	
Death, n (%)	3 (27.3%)	4 (36.4%)	1.000	3 (33.3%)	4 (30.8%)	1.000
ADL^¶^ in all, median (IQR)	14 (14-23.5)	14 (14-16)	1.000	14 (14-20.5)	14 (14-14)	0.070
PSM, median (IQR)	6 (6-6)	6 (6-6)	0.955	6 (6-10)	6 (6-6)	0.072
IADL, median (IQR)	8 (8-17.8)	8 (8-10)	1.000	8 (8-14.5)	8 (8-8)	0.070
TICS-M^¶^ (0-50), median (IQR)	35 (25-37.8)	36 (31-37)	0.771	29.5 (17-37)	35 (34-36)	0.678
Sequelaes^¶^, n (%)						
Symptomatic seizure, n (%)	2 (25.0%)	1 (14.3%)	1.000	2 (33.3%)	1 (11.1%)	0.525
NPSes, n (%)	1(12.5%)	3 (42.9%)	0.282	2 (33.3%)	2 (22.2%)	1.000
Cognitive impairment, n (%)	2 (25.0%)	2 (28.6%)	1.000	3 (50%)	1 (11.1%)	0.235

GABA, γ-aminobutyric acid; IQR, interquartile range; CSF, cerebrospinal fluid; OB, oligoclonal bands; MRI, magnetic resonance imaging; mRS, modified Rankin Scale; ADL, activities of daily living; PSM, physical self-maintenance; IAD, instrumental ADL; NPSes, Neuropsychiatric symptoms; TICS-M, modified telephone interview for cognitive status.

^¶^Those items were valued in 15 living patients

### Comparison of Outcomes Between Groups With High-Titer and Low-Titer Antibody Levels

Based on the antibody titers, the enrolled patients were divided into two groups: a high-titer group (9) and a low-titer group (13). The clinical characteristics and prognosis between the two groups are shown in **Table 3**. Overall, no significant differences were observed between the two groups in terms of demographics, clinical manifestations, main accessory examinations or functional outcomes.

## Discussion

We provide a comprehensive investigation in a relatively large cohort (22 patients) of rare AE in China. In evaluating neurological function and its influencing factors in patients with anti-GABA_B_R encephalitis, this study revealed several novel findings. (1) In patients with anti-GABA_B_R encephalitis, tumors occur more often in older patients, especially those with serum tumor markers and additional onconeuronal antibodies; and paraneoplastic patients are more likely to develop status epileptics, central hypoventilation and hyponatremia. (2) Although patients with tumors have a shorter time to immunotherapy initiation, they have a poor prognosis and high mortality. (3) After immunotherapy, although a majority of the patients without tumors achieved self-care, some of them still experienced neurological deficits.

It has been reported as paraneoplastic or idiopathic in case series of anti-GABA_B_R encephalitis, and LE patients with anti-GABABR antibodies are either with or without underlying tumors. Here, we showed a tumor rate of 36% with the vast majority of the neoplastic cases being SCLC, which is similar to the prevalence (33.3%-38.7%) of tumors in two studies in China (10, 12). However, as in other previous studies, approximately 50% of cases have an associated tumor (3, 8, 14, 15). We cannot exclude the possibility that tumors were detected after discharge in the four patients who were lost to follow-up. Moreover, inadequate follow-up or tumor screening may play a role, as not all patients in this cohort were followed up for more than four years or received PET/CT as a routine tumor screening method during the acute phase and the follow-up period.

In addition to anti-GABA_B_R antibody, three patients with SCLC had additional onconeuronal antibodies (to Hu in two patients and to CV2 in one patient). Because anti-Hu antibody recognize a family of RNA-binding proteins expressed in the nuclei of neurons and SCLC cells, it is highly associated with SCLC. As reported previously, it is the most frequently detected antibody in paraneoplastic seizures or status epilepticus (16). Lung tumor can be found in 77% of anti-Hu antibody positive patients with paraneoplastic encephalomyelitis/sensory neuropathy (17). Similarly, the most commonly reported autoantibodies associated with GABA_B_R autoimmune disease were anti-Hu (10.8%), anti-SOX1 (10.8%) and anti-GAD65 (8.5%), as summarized by a review of 94 cases with anti-GABA_B_R encephalitis (18). The coexistence of all these onconeuronal antibodies suggested autoimmunity caused by tumors, ie. an immune response against onconeuronal antibody expressing tumor cells that also destroy onconeuronal antibody expressing neurons. Therefore, if the patient shows GABA_B_R antibody along with other onconeuronal antibodies, SCLC should be considered until proven otherwise. In another study, level of serum pro-gastrin releasing peptide (ProGRP) was higher in anti-GABA_B_R encephalitis patients than in normal population, and the level of serum ProGRP showed significant difference between SCLC and non-SCLC subgroup (15). It is a pity that proGRP was not a routine test in our study. However, NSE as another tumor marker, was found to be more frequently in the patients of anti-GABA_B_R encephalitis with lung cancer. Although elevated NSE may be caused by severe brain injury and can be found in patients without tumor, it was detected more often in neoplastic cases, suggesting its potential as a tumor marker for SCLC in cases with anti-GABA_B_R encephalitis. Therefore, we infer the tests of serum paraneoplastic antibodies associated with GABA_B_R autoimmune disease and tumor markers associated with SCLC, which can initially suggest whether the patient has tumors (such as SCLC), in order to expect early detection of potential tumors and timely treatment.

In this study, the median age (60) of the paraneoplastic group was higher than that (52) of the group without tumors, suggesting that tumors occur more often in older patients. Interestingly, it was reported that when anti-GABA_B_R encephalitis occurs before the age of 45, there seems to be no association with cancer (3, 8, 19). Furthermore, paraneoplastic patients were more likely to develop status epileptics, central hypoventilation and hyponatremia in this study. Therefore, in elderly patients with probable AE with unknown etiology, especially who are accompanied by critically ill situations, testing for anti-GABA_B_R antibodies and suspicion of occult neoplasm is important.

Nonparaneoplastic anti-GABA_B_R encephalitis seems to better respond to immunomodulatory treatment and have a better prognosis. From this study, 31.8% (7/22) of patients had resistance to first-line immunotherapy, including 50% (4/8) of patients with tumors and 21.4% (3/14) of patients without tumors. The mortality rate of patients with tumor comorbidity was as high as 87.5%, suggesting that the prognosis of anti-GABA_B_R encephalitis with lung cancer was very poor. In a previous study, GABA_B_ receptor was also found to be expressed in lung cancer tissues of the patients diagnosed with anti-GABA_B_R encephalitis by immunohistochemistry (14). It hypotheses that ectopic expression of neuronal proteins by the tumor may lead to the reduction of immune tolerance for these proteins, which thus contributes to cancer-induced immune responses directed toward neuronal proteins and then the development of the autoimmune encephalitis. Early tumor resection and more aggressive upfront immunotherapy may improve the prognosis. In this cohort, deaths did not occur in the group without tumor comorbidity, which is inspiring. The results of the last follow-up of the 15 patients who survived showed that the patients could basically take care of themselves in their daily lives. However, instrumental activities of daily living were decreased, and the TICS-M suggested severe memory deficits in those patients. In previous report, Maureille et al. (11). retrospectively reported that two years after onset, massive anterograde amnesia affected all surviving patients with anti-GABA_B_R encephalitis in France. The lack of improvement in anterograde amnesia with treatment suggests neuronal death rather than functional impairment of synaptic transmission. One possible cause could be excitotoxicity due to extended SE (11), but the hypothesis of T cell mediated cytotoxicity could also be considered (11).

Previously, antibody titers against GABA_B_R in the serum ranged from 1:40 to 1:10,240 but did not correlate with disease severity (3). Consistent with that, no correlation between the antibody titers in CSF and disease severity was observed in this study. In addition, no correlation between the antibody titers in CSF and functional outcomes was found. Although there was a tendency for longer stays in the severe group in the hospital stay, there were no significant differences in functional outcomes between the nonsevere group and the severe group. Possible explanations are that bad functional outcomes were mainly caused by tumors or their complications.

The main limitation of this study was the small number of patients with anti-GABA_B_R encephalitis, and more data are needed to get more accurate statistics due to the lower incidence of anti-GABA_B_R encephalitis and fewer patients. The study was also subject to referral bias because the data were collected from a highly reputed national referral hospital known for neurology. In addition, the correlation between the immunotherapy and neurological outcomes is still uncertain, and the optimization of therapy needs to be further investigated. However, the findings from this study have several practical implications. The identification of elderly patients with anti-GABA_B_R antibodies, especially those with severe symptoms, serum tumor markers or additional onconeuronal antibodies, should prompt tumor screening with CT of the thorax/abdomen and PET/CT. Although patients with tumors had a shorter time to immunotherapy initiation, they had a higher mortality. After immunotherapy, although a majority of the patients without tumors achieved self-care, some of them were still experienced neurological deficits.

## Data Availability Statement

The raw data supporting the conclusions of this article will be made available by the authors, without undue reservation.

## Ethics Statement

The studies involving human participants were reviewed and approved by the Ethics Committee of Xuanwu Hospital, Capital Medical University (2020-104). The patients/participants provided their written informed consent to participate in this study.

## Author Contributions

WC designed and administrated the study, analyzed data and drafted paper. YW and XG participated within the investigation, data curation and analysis. LG, ZH, YL, QX, and GL took part in investigation. YZ and YS provided the resources, supervised the study and reviewed the manuscript. All authors contributed to the article and approved the submitted version.

## Funding

This study was supported by National Key Research and Development Program of China (No. 2020YFC2005403), Beijing Municipal Science & Technology Commission (No. Z211100002921030) and Beijing Municipal Administration of Hospitals Incubating Program (No. PX2020035).

## Conflict of Interest

The authors declare that the research was conducted in the absence of any commercial or financial relationships that could be construed as a potential conflict of interest.

## Publisher’s Note

All claims expressed in this article are solely those of the authors and do not necessarily represent those of their affiliated organizations, or those of the publisher, the editors and the reviewers. Any product that may be evaluated in this article, or claim that may be made by its manufacturer, is not guaranteed or endorsed by the publisher.
